# Transplantation of Wnt5a-modified Bone Marrow Mesenchymal Stem Cells Promotes Recovery After Spinal Cord Injury via the PI3K/AKT Pathway

**DOI:** 10.1007/s12035-024-04248-8

**Published:** 2024-05-25

**Authors:** Haimei Yang, Chaolun Liang, Junhua Luo, Xiuzhen Liu, Wanshun Wang, Kunrui Zheng, Dan Luo, Yu Hou, Da Guo, Dingkun Lin, Xiasheng Zheng, Xing Li

**Affiliations:** 1 State Key Laboratory of Dampness Syndrome of Chinese Medicine, Department of Orthopedic Surgery, The Second Affiliated Hospital of Guangzhou University of Chinese Medicine, Guangzhou, 510120 Guangdong China; 2 School of Pharmaceutical Sciences, Guangzhou University of Chinese Medicine, Guangzhou, 510006 Guangdong China; 3 Lingnan Medical Research Center of Guangzhou University of Chinese Medicine, Guangzhou, 510405 Guangdong China; 4 Department of Orthopedics (Joint Surgery), Guangdong Province Hospital of Chinese Medicine, Zhuhai, 519015 Guangdong China

**Keywords:** Wnt5a, Spinal cord injury, Bone marrow mesenchymal stem cells, Transplantation, Neuronal differentiation, PI3K/AKT pathway

## Abstract

Spinal cord injury (SCI) is a severe neurological condition that can lead to paralysis or even death. This study explored the potential benefits of bone marrow mesenchymal stem cell (BMSC) transplantation for repairing SCI. BMSCs also differentiate into astrocytes within damaged spinal cord tissues hindering the cell transplantation efficacy, therefore it is crucial to enhance their neuronal differentiation rate to facilitate spinal cord repair. Wnt5a, an upstream protein in the non-classical Wnt signaling pathway, has been implicated in stem cell migration, differentiation, and neurite formation but its role in the neuronal differentiation of BMSCs remains unclear. Thus, this study investigated the role and underlying mechanisms of Wnt5a in promoting neuronal differentiation of BMSCs both in vivo and in vitro. Wnt5a enhanced neuronal differentiation of BMSCs in vitro while reducing astrocyte differentiation. Additionally, high-throughput RNA sequencing revealed a correlation between Wnt5a and phosphoinositide 3-kinase (PI3K)/protein kinase B(AKT) signaling, which was confirmed by the use of the PI3K inhibitor LY294002 to reverse the effects of Wnt5a on BMSC neuronal differentiation. Furthermore, transplantation of Wnt5a-modified BMSCs into SCI rats effectively improved the histomorphology (Hematoxylin and eosin [H&E], Nissl and Luxol Fast Blue [LFB] staining), motor function scores (Footprint test and Basso-Beattie-Bresnahan [BBB]scores)and promoted neuron production, axonal formation, and remodeling of myelin sheaths (microtubule associated protein-2 [MAP-2], growth-associated protein 43 [GAP43], myelin basic protein [MBP]), while reducing astrocyte production (glial fibrillary acidic protein [GFAP]). Therefore, targeting the Wnt5a/PI3K/AKT pathway could enhance BMSC transplantation for SCI treatment.

**Supplementary Information:**

The online version contains supplementary material available at 10.1007/s12035-024-04248-8.

## Introduction

Spinal cord injury (SCI) is a significant neurological condition that can result from trauma, tuberculosis, tumors, and other factors [1]. Damage to the spinal cord tissue's structural integrity leads to impaired functioning characterized by sensory and motor deficits, reflex loss, and other dysfunctions. Secondary injuries such as inflammation and colloid scarring further worsen the condition, contributing to long-lasting impairments and increased disability and mortality rates in SCI patients [2, 3]. Although several treatment modalities including surgical decompression, pharmacotherapy, hypothermia, stem cell therapy, and growth factor therapy have been developed, their effectiveness remains limited due to the restricted self-repair mechanisms within the damaged neurological system [4].

Stem cell transplantation has emerged as a potential therapeutic approach for SCI, particularly bone marrow mesenchymal stem cells (BMSCs) that have considerable potential for nerve regeneration in SCI patients [5–7]. BMSCs can be readily obtained from the bone marrow and differentiate into neuronal cells, leading to improved motor function in SCI [8, 9]. However, an important limitation of BMSC therapy is their tendency to also differentiate into astrocytes within injured spinal cord tissues, impacting the overall efficacy of stem cell therapy [10–12].

Wnt proteins, a class of glycoproteins, exert significant effects on cell differentiation, proliferation, and migration. At least 19 Wnt proteins have been identified in humans and mammals and classified into traditional Wnt proteins (e.g., Wnt3a and Wnt7a) and non-classical Wnt proteins (e.g., Wnt5a and Wnt11) based on their downstream signaling pathways [13, 14]. Both classical and non-classical Wnt signaling pathways have been implicated in neuronal development [15–18] but the traditional Wnt/β-catenin pathway is inherently carcinogenic and susceptible to interference from extraneous signals, limiting its practical implementation and clinical application for neuronal repair [16, 19–23]. The non-classical protein Wnt5a [24] is involved in neurogenesis within the hippocampus and has implications for endogenous neural repair [25]. It facilitates the targeted differentiation of neural stem cells towards a neuronal lineage [26] and can enhance the differentiation or functionality of various stem cell types [27–30]. However, its potential role in BMSCs is unclear, therefore, this study investigated the potential of Wnt5a in promoting targeted neuronal differentiation of BMSCs both in vitro and in an animal model to provide supporting evidence for the application of Wnt5a in BMSC transplantation.

## Materials and Methods

### Animals

The Ethics Committee for Laboratory Animal Management at Guangzhou University of Chinese Medicine conducted a comprehensive evaluation and approved all animal research activities. The experimental protocol for this study is registered in The Second Affiliated Hospital of Guangzhou University of Chinese Medicine. Male Sprague-Dawley (SD) rats weighing 180-220g were obtained from the Experimental Animal Centre of Guangzhou University of Chinese Medicine. The rats were housed in specific rearing conditions, including a maximum cage occupancy of 5 rats, an ambient temperature ranging from 22 to 26°C, a 12-hour light-dark cycle, and a relative humidity of 55% to 68%. Before the experimental study, the rats were provided with appropriate nutrition for one week.

### The isolation and cultivation of BMSCs

Two-week-old male SD rats were euthanized using CO2. Under aseptic conditions, the tibia and femur were removed, cleansed, and sliced to expose the bone marrow cavity. The cavity was rinsed with phosphate-buffered saline (PBS) until whitened, and the eluate was collected and inoculated in glass dishes containing α-MEM medium (1X; Gibco, Life Technologies, USA), 10% fetal bovine serum (FBS; Gibco, Life Technologies, USA), and 100 U/mL antibiotics (Gibco, Life Technologies, USA). The cells were cultured at 37°C with 5% CO2, and the medium was changed every 3 days.

### Grouping and Treatment of BMSCs

The cultured BMSCs were seeded into 24-well plates (2×10^3^ cells per well) and divided into three groups: control, negative control (NC), and Wnt5a. The control group was maintained in the original medium, while the NC and Wnt5a groups underwent modified culture conditions to induce differentiation. Before differentiation, BMSCs were preconditioned in DMEM/F12 medium (1:1) to achieve a healthy state and allowed to adhere. The medium was then supplemented with 1% N-2 supplement CTSTM (100X; Gibco), 2% B27^TM^ supplement (50X; Gibco), 1% L-glutamine (Gibco), 20 ng/mL of brain-derived neurotrophic factor (BDNF; PeproTech, Rocky Hill, NJ, USA), 10 ng/ml epidermal growth factor (EGF; PeproTech), and 10 ng/ml basic fibroblast growth factor (bFGF; PeproTech without (NC group) or with Wnt5a (Wnt5a group) [31]. The medium was replaced every 3 days.

For the analysis of the phosphoinositide 3-kinase (PI3K) / protein kinase B (AKT) signaling pathway, BMSCs were cultured continuously for 15, 30, 60, and 120 minutes with or without Wnt5a. Then, 25μmol/L LY294002 (MedChemExpressly, China) was used to block the PI3K/AKT signaling pathway for 30 minutes. Subsequently, the cells were removed from the inhibitor environment and the cells continued to be cultured for 12 days to evaluate the related experiments.

### High-throughput RNA Sequencing (RNA-seq) and Bioinformatics Analysis

RNA was extracted using TRIzol reagent (Invitrogen, CA, USA) in accordance with the provided instructions. The purity, quantity, and integrity of the RNA were subsequently assessed. Subsequently, cDNA libraries were constructed using the VAHTS Universal V6 RNA-seq Library Preparation Kit (Vazyme Biotech, China) according to the manufacturer's guidelines. Sequencing was performed on the Illumina Novaseq 6000 platform, resulting in the generation of double-ended read sequences with a length of 150 base pairs. After removing low-quality data, bioinformatics analysis was performed.

Sequence alignments were executed to ascertain similarities among the samples. Concurrently, the Fragments Per Kilobase of transcript per Million mapped reads (FPKM) metric was calculated utilizing the HISAT2 software. Principal Component Analysis (PCA) was used in R version 3.2.0 to evaluate the biological consistency of the data. Additionally, differential expression analysis was performed using the DESeq2 package. To identify differentially expressed genes (DEGs), a significance threshold of Q value < 0.05 and a fold change greater than 2 or less than 0.5 were used for screening. Subsequent to the differential gene expression analysis, hierarchical clustering analysis and KEGG pathway enrichment analysis were performed using the R program.

### Lentivirus Construction and Transfection

Lentiviral vectors, including the Wnt5a overexpressing lentiviral vector (Wnt5a-LV) and the empty lentiviral vector (NC-LV), were constructed. Lentiviral vectors for Wnt5a overexpression were constructed using rat DNA as a template, and amplification primers for Wnt5a were designed according to NCBI. Subsequently, 293T cells were co-transfected with the vector and packaging plasmid. After 8 hours of culture, the cells were transferred to complete medium for 48 hours. The supernatant was collected, filtered, concentrated, and resuspended. The optimal MOI was determined via titer assay. BMSCs were infected with the two lentiviruses, resulting in NC-BMSCs (NC group) and Wnt5a-BMSCs (Wnt5a group). Overexpression of Wnt5a was assessed by western blotting analysis.

### Western Blotting Analysis

Total protein from BMSCs was extracted using ristocetin-induced platelet aggregation (RIPA) buffer (Gibco, Grand Island, NY, USA) containing a phosphatase inhibitor (1nM Na_3_VO_4_ and 1nM NaF) and a protease inhibitor (1 μg/ml; Sigma-Aldrich). The protein content was quantified using the BCA protein assay reagent [32]. Next, total protein per group (20 μgg) was loaded onto a 10% sodium dodecyl sulfate polyacrylamide gel electrophoresis (SDS-PAGE) and then transferred to a PVDF membrane. The membrane was sealed using pre-chilled NcmBlot Rapid Closure Solution, and primary antibodies were added at 4°C: microtubule-associated protein-2 (MAP-2) (1:1000; Boster Biological Engineering Co.), glial fibrillary acidic protein (GFAP) (1:1000; Boster Biological Engineering Co.), β3-tubulin (1:1000; CST), growth-associated protein 43 (GAP43) (1:1000; NOVUS), myelin basic protein (MBP) (1:1000; NOVUS), PI3K (1:1000; CST, 4228), p-PI3K (1:1000; Bioss), AKT (1:1000; CST), p-AKT (1:1000; CST), and glyceraldehyde-3-phosphate dehydrogenase (GAPDH) (1:500; Thermo Fisher). After rinsing the membranes with Tris-buffered saline with Tween (TBST), they were incubated for 60 minutes with a secondary antibody (1:1000; Boster Biological Engineering Co.). Proteins were visualized using a ChemiDoc^TM^ MP imaging system (Bio-Rad). The relative intensity of each band was measured using Image J (National Institutes of Health, Bethesda, MD). The relative intensity of the p-PI3K versus p-AKT bands was determined using the PI3K and AKT proteins, respectively. Additionally, the entire phosphorylated protein assay was conducted at a temperature of 4°C.

### Establishment of the Rat Model of SCI

The rat model of SCI was established using Allen's method [33]. The rats were immobilized under aseptic conditions with general anesthesia, and their back skin was shaved. An incision (3 cm) was made with the spinal cord of the T10 segment as the center to expose the spinous processes and plates of the T9-T11 segments. The spinous processes and plates were removedto fully expose the spinal cord tissues. The T10 spinal cord was impacted using a PinPoint^TM^ precision SCI impinger (striking speed 1.2 m/s; striking depth 1.0 mm; stopping time 85 ms). It was observed that the struck portion of the spinal cord rapidly congested and reddened, and the rats exhibited transient spastic convulsions in their tail and hind limbs, indicating successful construction of the SCI model. In the sham-operated group, the spinal cord tissue was exposed without impact.

### Grouping and Treatment of SCI Model

Twenty-four rats were randomly divided into four groups (n=6): Sham group, SCI group, NC group, and Wnt5a group. Cell transplantation was performed through tail vein injection. Three days after the surgery, rats in the Sham and SCI groups received a saline injection (1 ml) while those in the NC group were injected with NC-BMSCs single-cell suspension (1 ml, 2×10^6^ cells/ml), and rats in the Wnt5a group were injected with Wnt5a-BMSCs single-cell suspension (1 ml, 2×10^6^ cells/ml).

### Animal Behavioral Assessment

The behavioral assessment of SCI rats in each group was performed using the Basso-Beattie-Bresnahan (BBB) scale and footprint experiment [34, 35]. On the 3rd, 7th, 14th, and 21st days following BMSCs transplantation, the rats were placed in an open field for 15 minutes to assess hind limb motor ability using BBB scale, with scores ranging from 0 to 21 (0 represents complete paralysis of the hind limbs, and 21 represents normal hind limb movement). The rat hindlimb footprint experiment was conducted on the 21st day: the hind paws were dyed and placed on a 100 cm white paper-covered track. The rats were guided to the finish line to observe and record their locomotion and coordination.

### Tissue Preparation and Preservation

On the 21st day after BMSCs transplantation, all rats were euthanized. A section of the injured spinal cord tissue was preserved in liquid nitrogen for protein blotting analysis. Another portion of the tissue was decalcified in 4% paraformaldehyde (PFA) at room temperature for 30 days, dehydrated, embedded in paraffin, and sectioned to a thickness of 5 μm for histopathological staining.

### Histopathological Staining

Hematoxylin and eosin (H&E), Nissl and Luxol Fast Blue (LFB) staining were performed on the diseased tissue according to the manufacturer's instructions to observe the histopathological changes, including the number of neurons, morphology of the spinal cord tissue and integrity of the neuronal myelin sheath after SCI.

### Immunofluorescence Staining and Analysis

The cells were fixed in 4% paraformaldehyde (PFA) and permeabilized with 0.3% Triton X-100 at room temperature for 60 minutes. Tissue sections underwent deparaffinization sequentially with xylene and different concentrations of alcohol. Subsequently, the sections were treated with an antigen repair solution (10 mM sodium citrate, pH 6.0) for repair. After 1 hour of incubation with 10% goat serum, the following primary antibodies were added and incubated overnight at 4°C: GFAP antibody (1:600; Boster Bioengineering), MAP2 antibody (1:200; Boster Bioengineering), β3-tubulin antibody (1:200; Cell Signaling Technology), GAP43 antibody (1:200; NOVUS), and MBP antibody (1:200; NOVUS). The next day, DAPI was used to stain the nuclei, and cells were incubated with Alexa Fluor secondary antibody (1:300; Invitrogen) diluted in PBS corresponding to the primary antibodies. Finally, the fluorescent images were observed under a fluorescence microscope.

### Statistical Analysis

Statistical analyses were performed using SPSS version 16.0 (SPSS Inc., Chicago, IL, USA). Data were presented as mean ±standard error of measurement (SEM). Differences between groups were determined using one-way analysis of variance (ANOVA) or Student's t-test. A P value less than 0.05 was considered statistically significant.

## Results

### Wnt5a Promoted Neuronal Differentiation of BMSCs

**Fig. 1:**
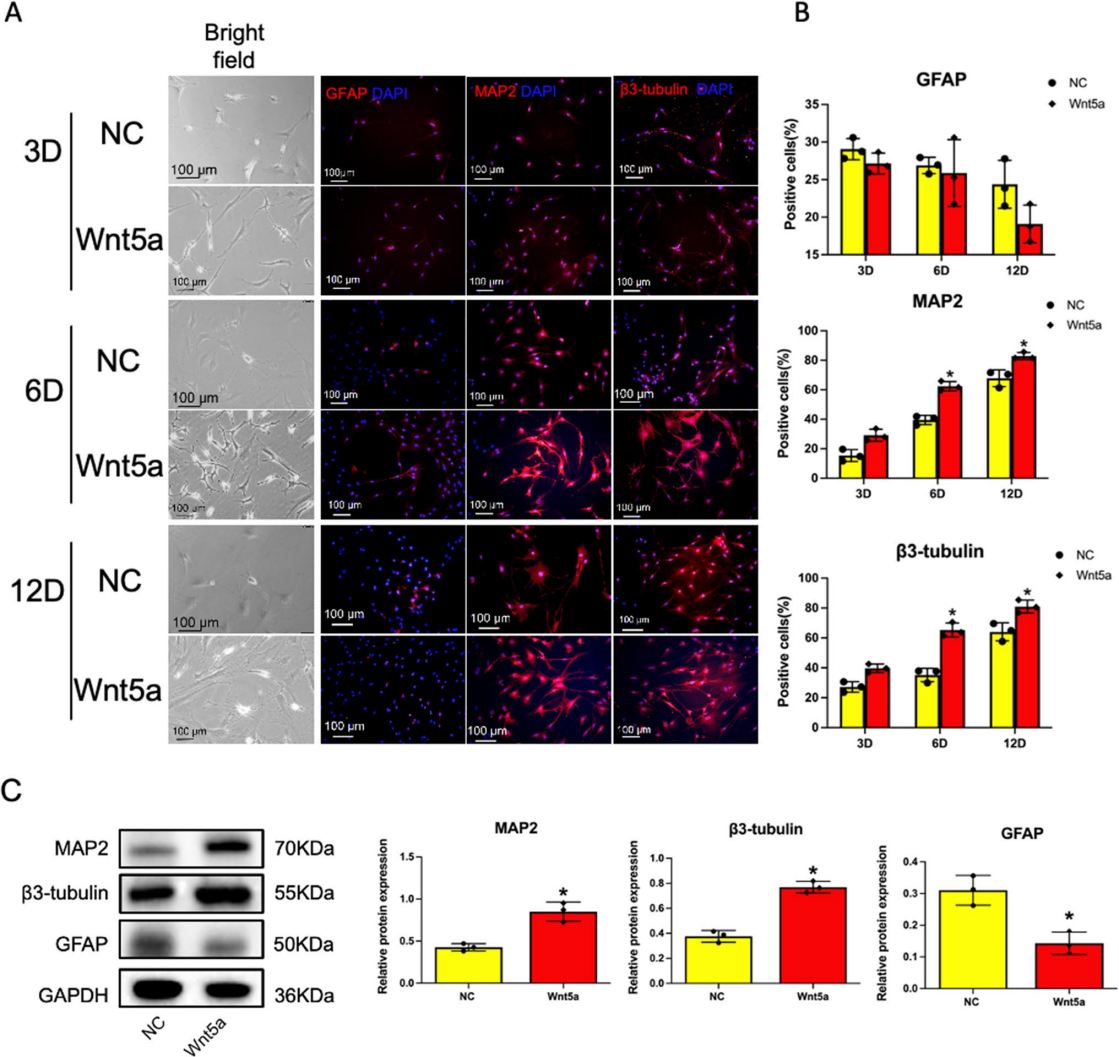
Modification of Wnt5a promotes BMSC differentiation into neurons and inhibits BMSC differentiation into astrocytes. **A**: Bright field and immunofluorescence staining on days 3, 6, and 12 of induced neuronal differentiation of Wnt5a-BMSCs using BMSCs unmodified with Wnt5a as a control group. **B**: The number of neural differentiation marker-positive BMSCs over time. Data are presented as mean ± SEM. *P<0:05 compared with the NC group. **C**: Comparison of MAP2, β3-tubulin, and GFAP expression in the NC group versus the Wnt5a group measured by western blotting. Data are presented as mean ± SEM. *P<0:05 compared with the NC group

To assess the efficacy of Wnt5a in promoting neuronal differentiation in BMSCs, in vitro experiments were conducted. Initially, successful Wnt5a overexpression was confirmed (Appendix Figure). Subsequently, the results revealed an increase in both MAP2-positive and β3-tubulin-positive cells after 12 days of induction, as well as a decrease in GFAP-positive cells in both the Wnt5a and NC groups. Moreover, the changes were more significant in the Wnt5a group (*p<0.05*). In terms of cell morphology, cells in the Wnt5a group were more similar to neurons (Fig. 1A, B). These findings indicated that BMSCs treated with Wnt5a had enhanced neuronal differentiation, while the number of astrocytes in the differentiated cells decreased correspondingly. Furthermore, a 12-day protein blot analysis (Fig. 1C) provided additional support for this conclusion.

### BMSC Transcriptomic Sequencing Analysis

**Fig. 2:**
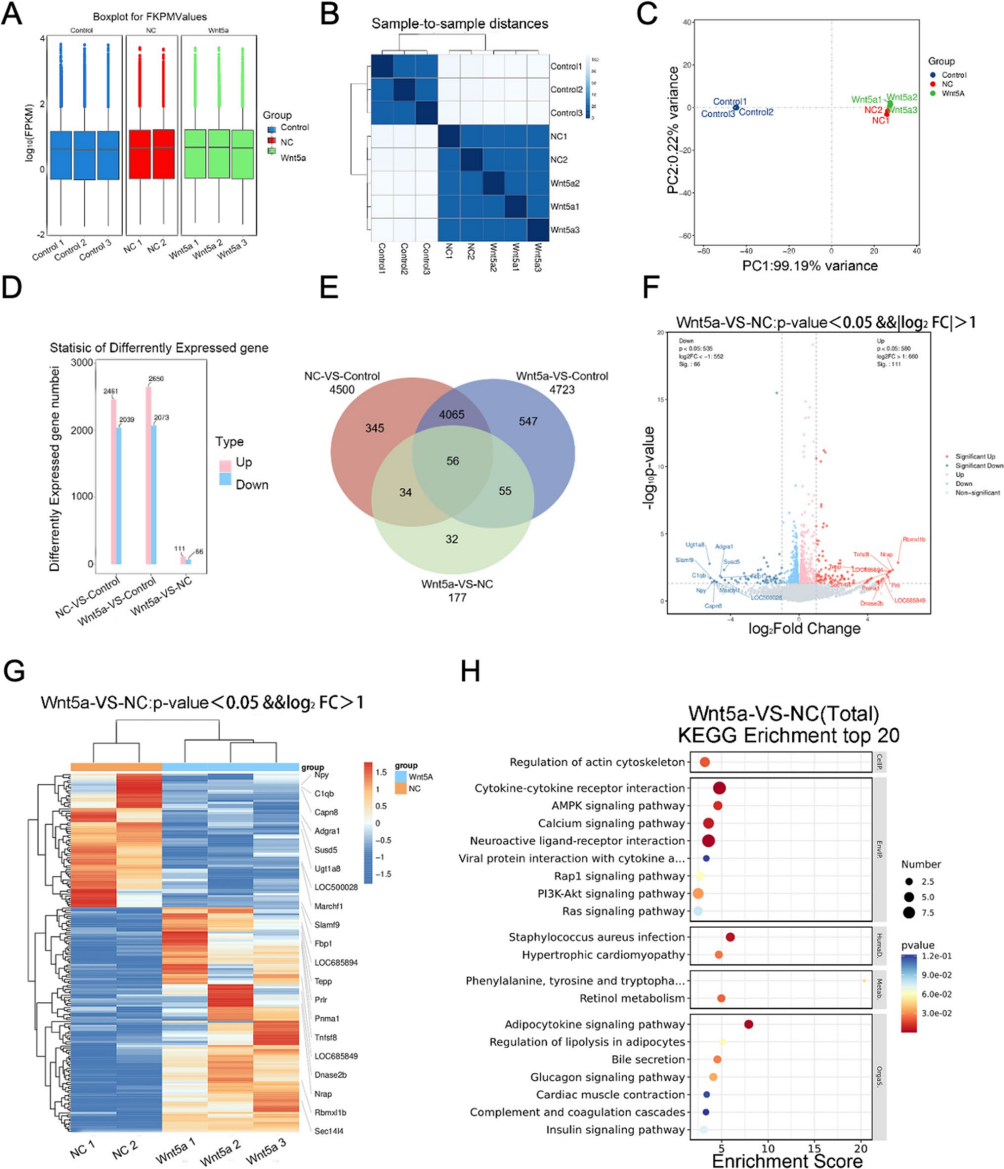
Transcriptomic sequencing results of BMSCs. **A**: The gene expression in the Control, NC, and Wnt5a groups was relatively consistent. **B**: The similarity of expression was higher for samples within groups and lower for samples between groups. **C**: Principal component analysis (PCA) revealed significant differences between the Wnt5a and NC groups. **D**: Differential metabolites in the Control, NC, and Wnt5a groups. Up is the number of significantly different up-regulated genes and down is the number of down-regulated genes. **E**: A Venn diagram depicting the common differentially expressed genes in the NC, Wnt5a, and Control groups. **F**: Volcano plot of differentially expressed genes in the Wnt5a versus the NC group. Blue dots represent downregulated genes and red dots represent upregulated genes. **G**: Clustering heatmap of differentially expressed genes in the Control, NC, and Wnt5a groups. The color scale illustrates the relative abundance of the samples with blue indicating significant down-regulation of metabolites and red indicating significant up-regulation of metabolites. **H**: KEGG enrichment analysis of differentially expressed genes in the Wnt5a and NC groups

High-throughput RNA sequencing was performed on samples from the control, NC, and Wnt5a groups. FPKM expression values were normalized for subsequent analysis (Fig. 2A). Similarity analysis and PCA principal component analysis revealed high intra-group similarity and low inter-group similarity, indicating the reliability and reproducibility of the experimental samples (Fig. 2B, C). DEG analysis identified 111 up-regulated and 66 down-regulated genes in the Wnt5a group compared to the NC group (Fig. 2D). Furthermore, 56 genes showed significant changes in expression across all three groups (Fig. 2E). Using log_2_FC absolute value > 1 and p < 0.05 as the screening criterion, further analysis identified 77 genes (11 up-regulated and 66 down-regulated) with significant differences between the Wnt5a and NC groups (Fig. 2F, G). KEGG enrichment analysis indicated that DEGs in the Wnt5a group primarily involved in cytological processes such as the regulation of the actin cytoskeleton and cytokine-receptor interactions. The involved pathways included the AMPK pathway and calcium pathway, and so on. Notably, the PI3K/AKT signaling pathway was closely associated with stem cell differentiation (Fig. 2H).

### Wnt5a Stimulated Neuron-directed Differentiation of BMSCs via the PI3K/AKT Pathway

**Fig. 3:**
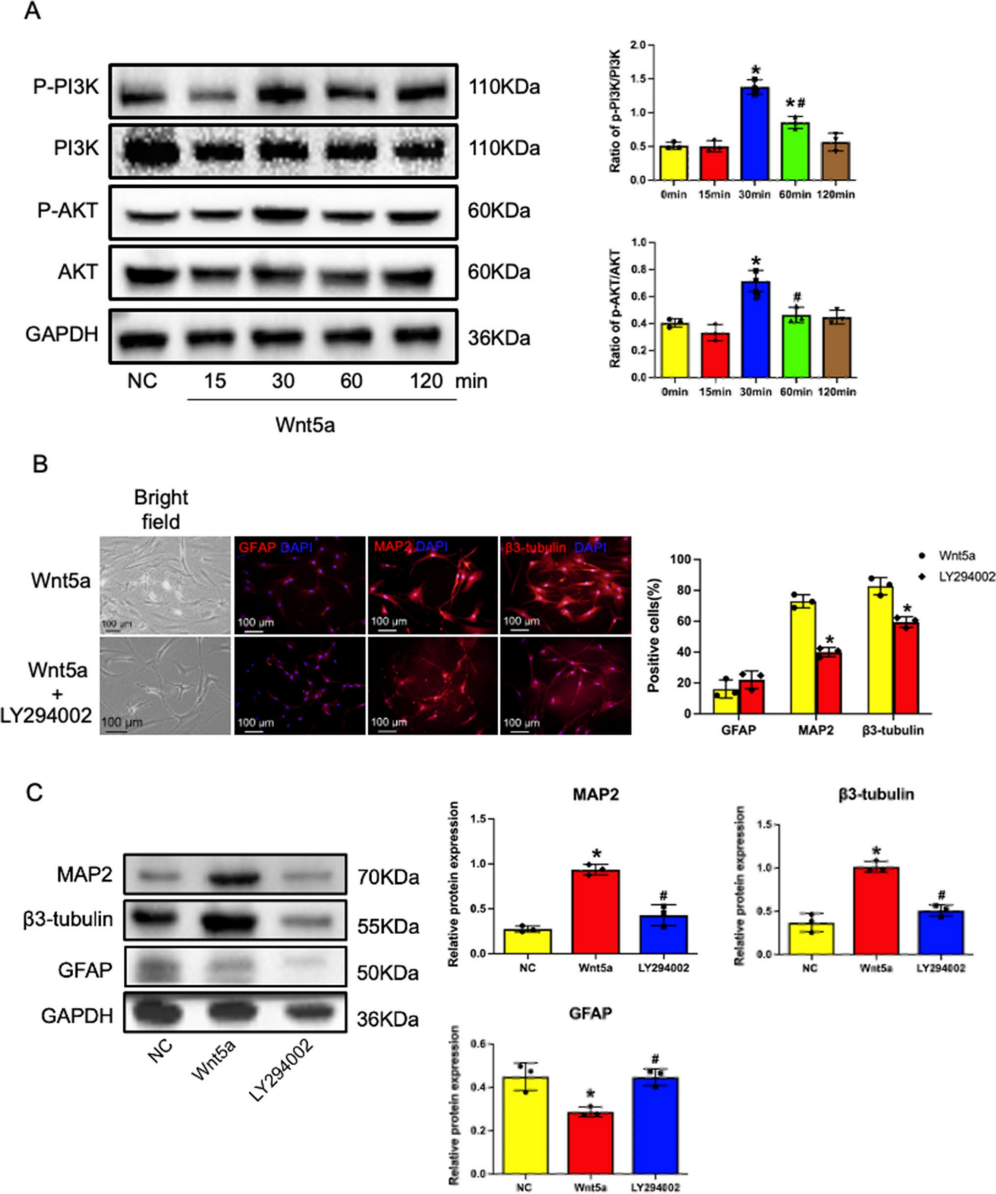
Wnt5a promotes neuronal differentiation in BMSCs through the PI3K/AKT signaling pathway. **A**: Time to phosphorylation activation of PI3K and AKT. Data are presented as mean ± SEM. *P<0:05 compared with 0 minute; #*P*<0:05 compared with 30 minute. **B**: The addition of the PI3K inhibitor LY294002 significantly decreased the percentage of neuronal marker-positive BMSCs (with no PI3K inhibitor as control). Data are presented as mean ± SEM. *P<0:05 compared to the Wnt5a group. **C**: Effects of PI3K inhibitors on MAP2, β3-tubulin, and GFAP expression in Wnt5a-modified BMSCs. Data are presented as mean ± SEM. *P<0:05 compared to the NC group; #*P*<0:05 compared to the Wnt5a group

To explore the involvement of the PI3K/AKT signaling pathway in Wnt5a-mediated promotion of neuron-directed differentiation of BMSCs, the phosphorylation of PI3K and AKT in cells from the NC and Wnt5a groups was examined. The results revealed increased phosphorylation of PI3K and AKT after 30 minutes (Fig. 3A). To further confirm the significance of the PI3K/AKT pathway, the PI3K inhibitor (LY294002) was used. The results demonstrated a substantial decrease in MAP2-positive and β3-tubulin-positive cells and protein after treatment with the PI3K inhibitor LY294002 (Fig. 3B, C) (*p<0.05*). These findings suggest that Wnt5a promotes neuronal differentiation of BMSCs through the PI3K/AKT signaling pathway.

### In vivo Transplantation of Wnt5a-BMSCs Improved Hindlimb Function in SCI Rats

**Fig. 4:**
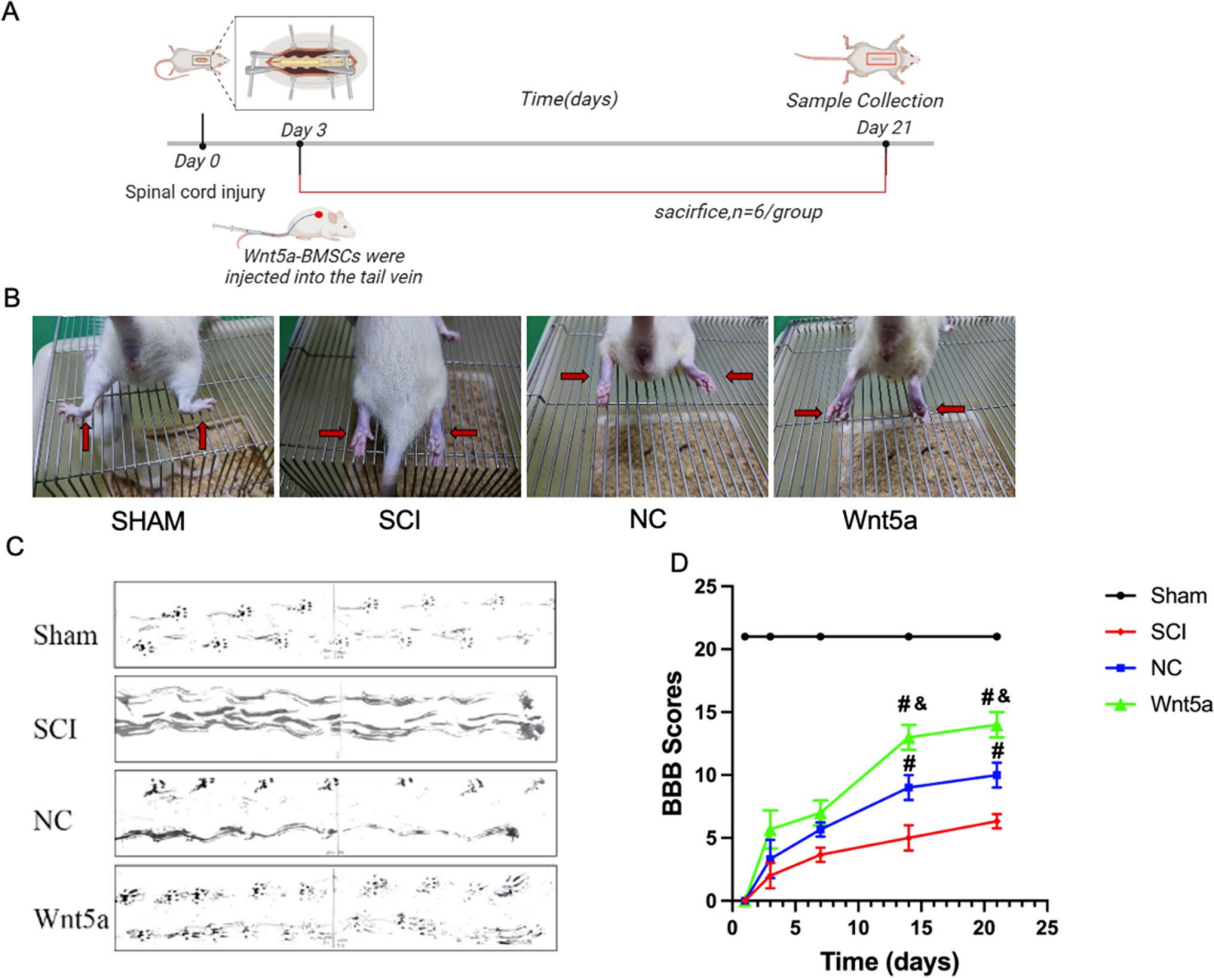

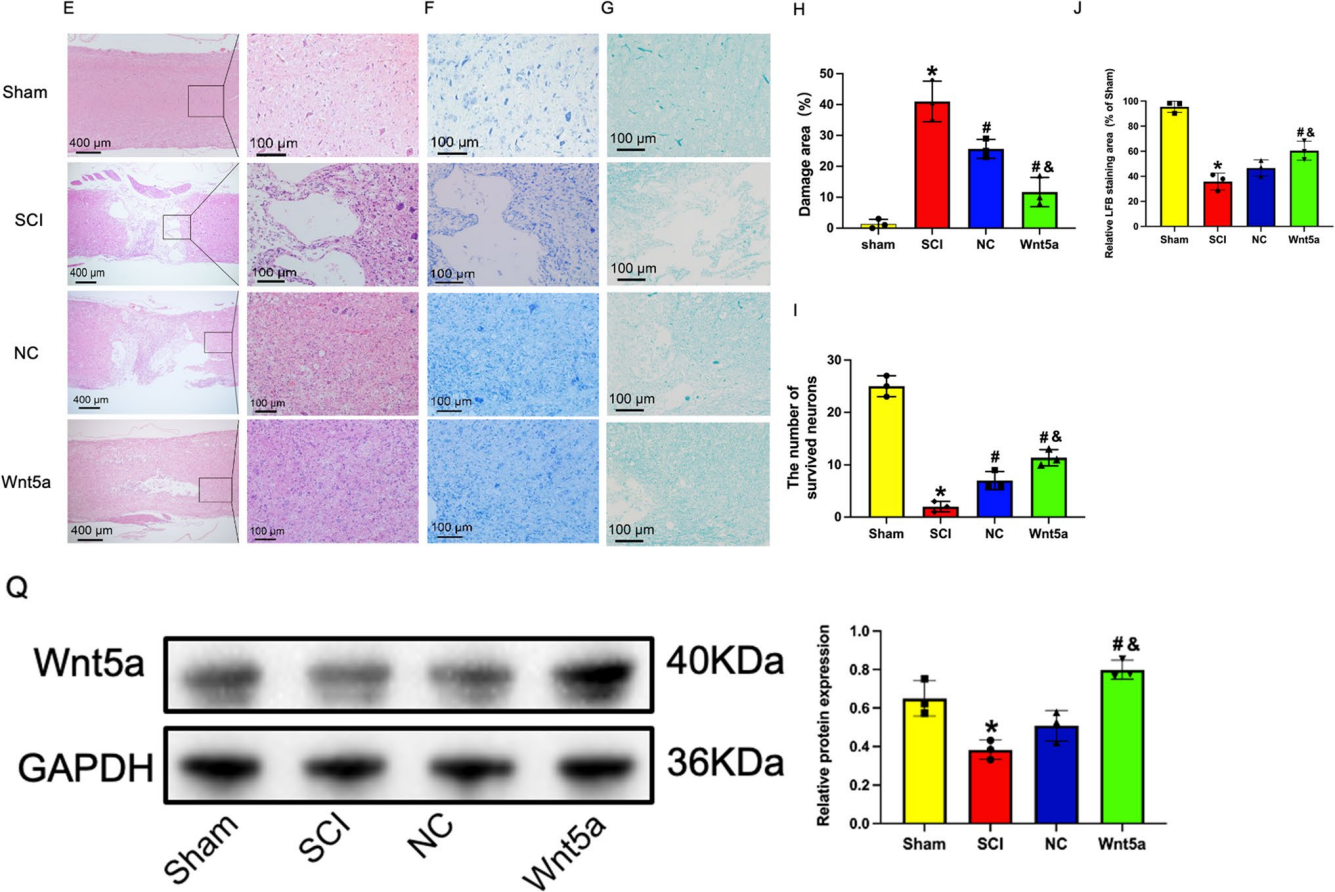
In vivo transplantation of Wnt5a-modified BMSCs promotes tissue repair and recovery of hindlimb motor function in SCI rats. **A**: Functional recovery test in SCI rats. **B**: Hindlimb status of SCI rats. **C**: Changes in hindlimb motor function in SCI rats were evaluated by inked footprint analysis. **D**: Motor function scores of rats. Data are presented as mean ± SEM. *#*P*<0:05 compared to the SCI group; &*P*<0:05 compared to the NC group. **E**: HE staining of SCI rat spinal cord tissue. **F**: Nissl staining of SCI rat spinal cord tissue. **G**: LFB staining of SCI rat spinal cord tissue. **H**: The damaged area of spinal cord tissues. Data are presented as mean ± SEM. *P<0:05 compared to the Sham group; #*P*<0:05 compared to the SCI group; &*P*<0:05 compared to the NC group. **I**: Surviving neurons in the spinal cord tissues. Data are presented as mean ± SEM. *P<0:05 compared to the Sham group; #*P*<0:05 compared to the SCI group; &*P*<0:05 compared to the NC group. **J**: Relative LFB staining area in the spinal cord tissues. Data are presented as mean ± SEM. *P<0:05 compared to the Sham group; #*P*<0:05 compared to the SCI group; &*P*<0:05 compared to the NC group. **Q**: Wnt5a protein expression in spine tissue samples was measured by Western blot. Data are presented as mean ± SEM. &*P*<0:05 compared to the Sham group; #*P*<0:05 compared to the SCI group; *P<0:05 compared to the NC group

To evaluate the efficacy of Wnt5a-transfected BMSCs in *vivo*, a SCI rat model was firstly created and the expression of Wnt5a in spinal cord tissues were determined (Fig. 4A, B, Q). Then the rats’ hindlimb function was assessed using the footprint experiment and the BBB scale. Footprint tests revealed normal footprints in the Sham group and a dragging pattern in the SCI group. Transplantation of NC-BMSCs resulted in partial improvement of locomotion in the left hind limb of SCI rats, which was further enhanced by transplantation of Wnt5a-BMSCs (Fig. 4C). The BBB scale confirmed these findings, showing significantly higher scores in both the NC and Wnt5a groups compared to the SCI group at 21 days, with a more pronounced advantage observed in the Wnt5a group. This advantage was evident as early as day 3 (*p<0.05*) (Fig. 4D). These results indicate that Wnt5a enhances the effectiveness of BMSCs in rat model.

### In vivo Transplantation of Wnt5a-BMSCs Promoted Spinal Cord Tissue Repair in SCI Rats

To determine the role of Wnt5a in promoting BMSC function for spinal cord tissue repair, histopathological changes in the spinal cords of SCI rats were examined. H&E staining results showed that the Sham group had numerous cells with normal morphology and a dense distribution among them. Following surgery, the SCI, NC, and Wnt5a groups exhibited a significant decrease in cell number and numerous large cavities in the spinal cord tissues. After BMSC transplantation, the damaged area in the spinal cord tissues reduced significantly in both transplantation groups, and the number of neurons with normal morphology also increased significantly (*p<0.05*). Moreover, rats transplanted with Wnt5a-BMSCs had better spinal cord tissue integrity and cellular status compared to those transplanted with NC-BMSCs (*p<0.05*). These findings were further supported by Nissl staining results. The results of LFB staining showed extensive myelin destruction in the tissues of SCI rats, whereas transplantation of BMSC significantly promoted the repair of myelin (the effect was significantly better in the Wnt5a group than in the NC group) (Fig. 4E, F, G, H, I, J). This suggests that Wnt5a facilitates the repair of damaged spinal cord tissues by BMSCs in SCI rats.

### Transplantation of Wnt5a-modified BMSCs in vivo Inhibited Astrocyte Generation and Stimulates Neuronal Regeneration

**Fig. 5:**
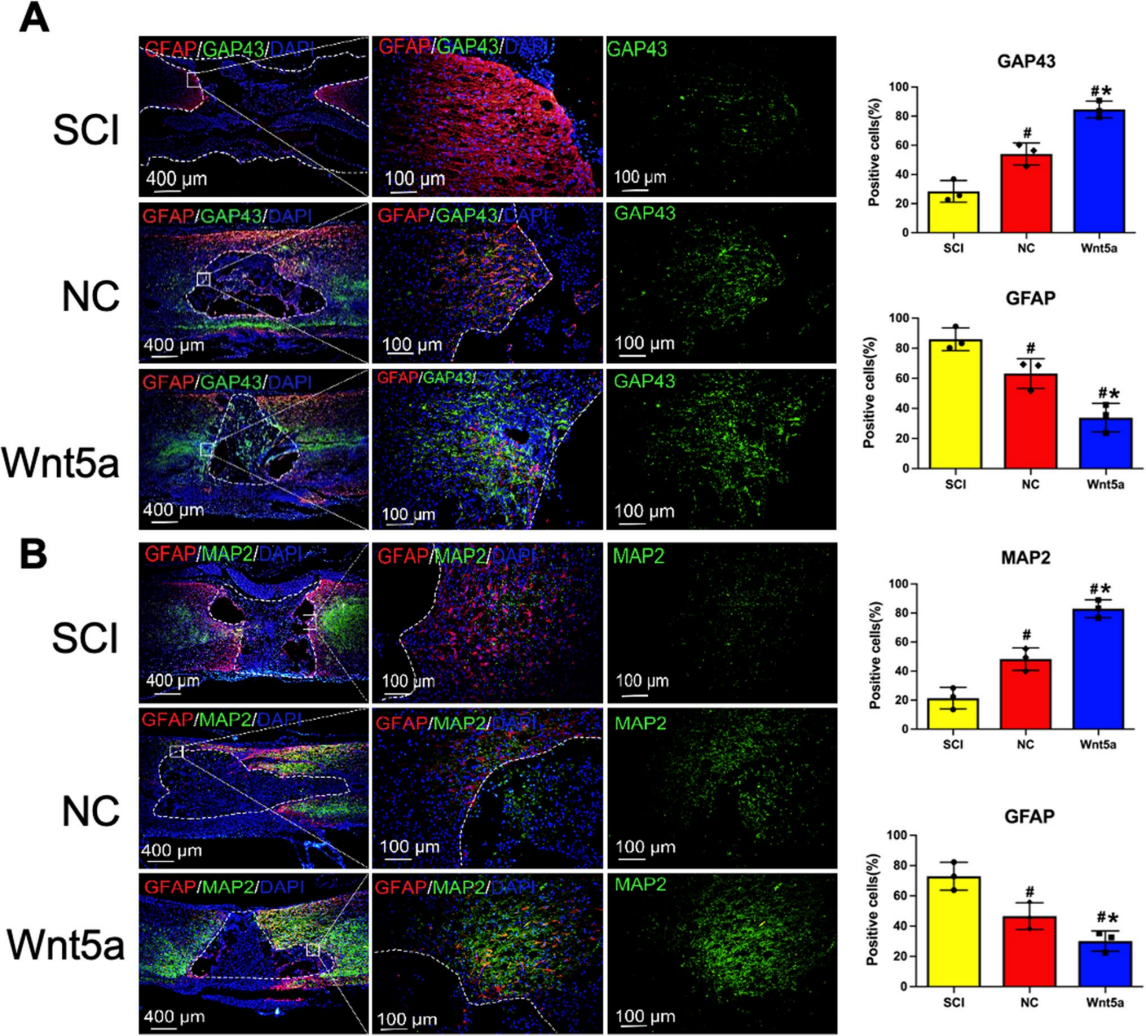

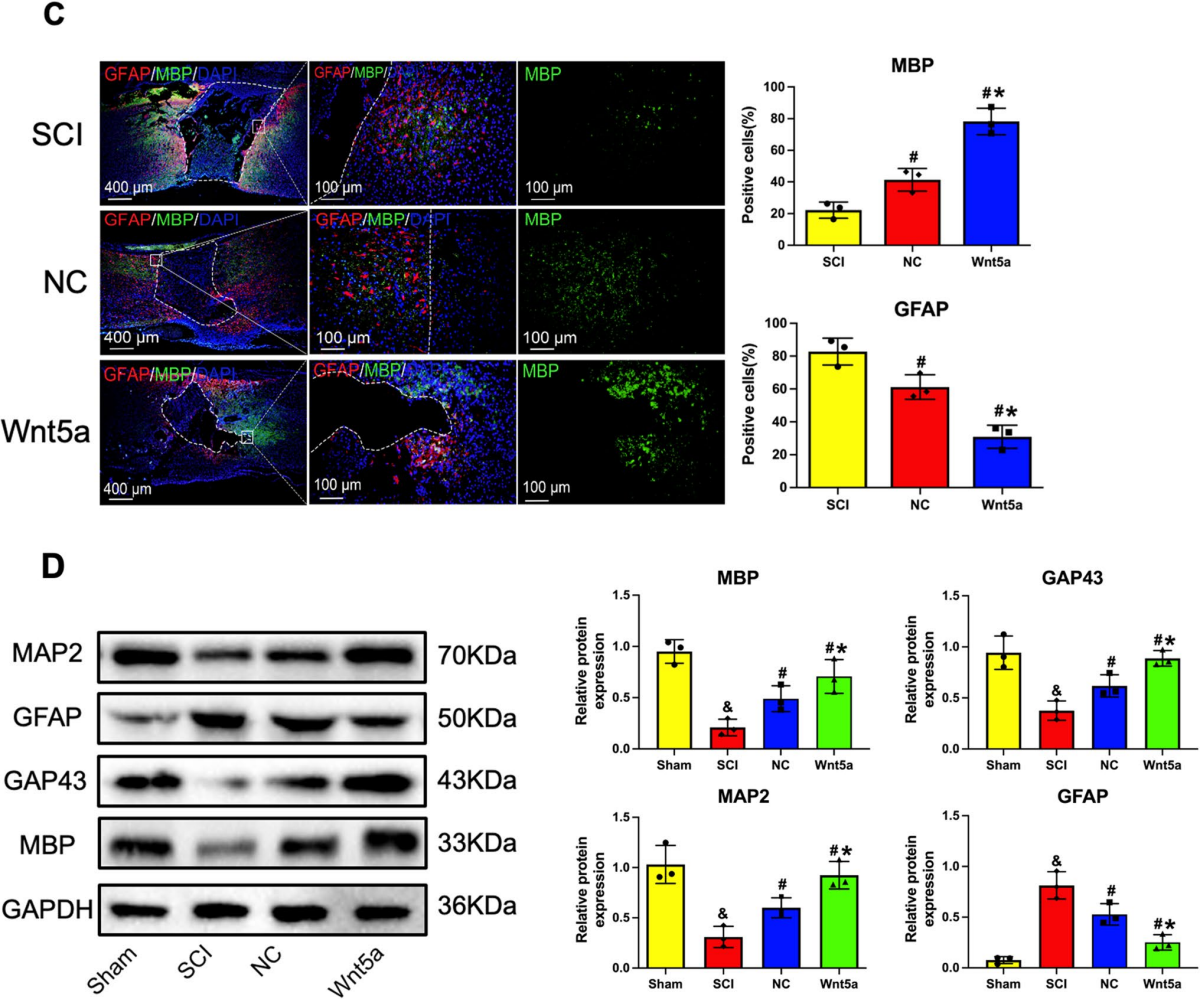
In vivo transplantation of Wnt5a-modified BMSCs reduces astrocyte production and promotes neuronal regeneration. **A**-**C**: Immunofluorescence staining of GAP43, MAP2, and MBP-positive cells in spinal cord tissues. Data are presented as mean ± SEM. #*P*<0:05 compared to the SCI group; *P<0:05 compared to the NC group. **D**: GAP43, MAP2, GFAP, and MBP protein expression were measured in spine tissue samples by western blotting. Data are presented as mean ± SEM. &*P*<0:05 compared to the Sham group; #*P*<0:05 compared with SCI group; *P<0:05 compared to NC group

To further investigate the effects of Wnt5a-modified BMSCs on astrocytes and neurons in vivo, immunofluorescence staining was conducted using markers GFAP, GAP43, MAP2, and MBP. GAP43 regulates axon growth and new junction formation, while MAP2 is involved in microtubule assembly and maintaining cellular structural integrity in mature neurons. MBP serves as an essential marker for myelin regeneration. Staining analysis revealed a significantly higher number of GAP43-positive, MAP2-positive, and MBP-positive cells in the BMSC-transplanted group compared to the SCI group (*p<0.05*). Furthermore, the transplantation of Wnt5a-modified BMSCs resulted in an even more significant increase in positive cells (*p<0.05*) (Fig. 5A-C). The protein blotting results were consistent with the fluorescent staining outcomes (Fig. 5D). Thus, transplantation of Wnt5a-modified BMSCs promotes the development of mature neurons, axon formation, and myelin remodeling.

GFAP staining, which serves as a significant indicator for astrocytes, revealed a substantial number of GFAP-positive cells in the SCI group. This number significantly decreased after the transplantation of unmodified BMSCs by Wnt5a (*p<0.05*) and further decreased after the transplantation of Wnt5a-modified BMSCs (*p<0.05*) (Fig. 5A-C). The protein blotting results were consistent with the fluorescent staining outcomes (Fig. 5D). This result demonstrated that BMSC transplantation substantially decreased astrocyte production, and Wnt5a modification further reduced astrocytes.

## Discussion

SCI is a severe neurological condition that often leads to permanent neurological dysfunction, imposing a significant burden on patients and their families [4, 36]. Currently, there is no clinically recognized and unequivocally effective treatment for SCI but stem cell differentiation into neurons to restore damaged spinal cord tissue has emerged as a promising therapeutic approach [37]. However, a considerable proportion of transplanted stem cells differentiate into astrocytes instead of neurons, reducing the efficacy of stem cell therapy [38–40]. Therefore, increasing the rate of neural differentiation following stem cell transplantation is crucial.

The Wnt signaling pathway in multicellular eukaryotes regulates various cellular processes such as cell proliferation, differentiation, migration, polarization, and modulation. It exerts significant effects on the recovery of neurological functions following central nervous system injury. Previous research has shown that both the classical Wnt/ β-Catenin pathway and non-classical Wnt pathways, including Wnt/JNK and Wnt/Ca^2+^, are associated with neuronal differentiation in stem cells [17, 41–44]. Wnt5a is an activator of the non-classical Wnt pathway and has been closely associated with neuron generation [25, 26, 45]. Furthermore, Wnt5a promotes the osteogenic differentiation of BMSCs [46–49]. However, few studies have examined its ability to promote neuronal differentiation of stem cells. Our preliminary investigation using high-throughput sequencing (Appendix 1) revealed the potential significance of Wnt5a in BMSC neuronal differentiation. Subsequently, *in vitro* assays were conducted to validate Wnt5a's involvement in facilitating neuronal differentiation induction and reducing the astrocyte count in BMSCs, as Wnt4, Wnt5a, and Wnt11 genes are upregulated in neurogenic induced-human bone marrow-derived mesenchymal stem cells [50].

The mechanism by which Wnt5a stimulates neuronal differentiation is unclear. Wnt5a-induced upregulation of miRNA200b-3p inhibits RhoA/Rock signaling pathway activation promoting neuronal differentiation of neural stem cells [26] but conversely, IL-1β stimulated Wnt5a activates the RhoA/Rock signaling pathway to facilitate the neuronal differentiation of neural precursor cells [17]. These results suggest that the Wnt5a signaling pathways are dependent on the cell type and its microenvironment. High-throughput RNA sequencing and KEGG pathway enrichment analyses revealed a connection between the PI3K/AKT pathway and Wnt5a-induced neuronal differentiation in BMSCs (Appendix 2), which was confirmed by the use of the PI3K inhibitor LY294002 to reduce the number of differentiated neurons from Wnt5a-modified BMSCs. The PI3K/AKT pathway exerts crucial effects on the regulation of neuronal cell growth, proliferation, and differentiation [51–53]. Previous studies have shown that Wnt5a affects the proliferation and differentiation of mesenchymal stem cells (MSCs) through the PI3K/AKT pathway [54, 55]. Additionally, we observed that Wnt5a controls neural differentiation of BMSCs via PI3K/AKT. PI3K/AKT/JNK activation of Wnt5a enhanced the differentiation and proliferation of MSCs and chondrocytes [56] and PI3K inhibition hampered the promotion of trigeminal ganglion neurite growth by Wnt5a [57]. Therefore, we hypothesized that Wnt5a/PI3K/AKT stimulates neural differentiation in BMSCs, potentially facilitating BMSC transplantation for SCI.

Assessments of rat motor performance and histology demonstrated that the introduction of Wnt5a-modified BMSCs enhances neuronal development and reduces the number of astrocytes. This was further supported by the identification of markers associated with neurons and astrocytes in spinal cord tissues, consistent with previous research findings [45]. LINGO-1, for example, enhances neuronal differentiation in neural stem cells and inhibits astrocyte differentiation through Wnt5a in rats. This study was the first to investigate the effect of Wnt5a on promoting neuronal differentiation of BMSCs for functional recovery following SCI, confirming Wnt5a's involvement in inducing neuronal differentiation while reducing astrocytes in BMSCs. Furthermore, we established a relationship between Wnt5a and the PI3K/AKT pathway, demonstrating that Wnt5a promotes targeted neuronal differentiation of BMSCs and improves SCI-related manifestations in an animal model. These findings support the potential efficacy of Wnt5a-BMSC transplantation as a promising therapeutic approach for SCI.

## Conclusion

**Fig. 6:**
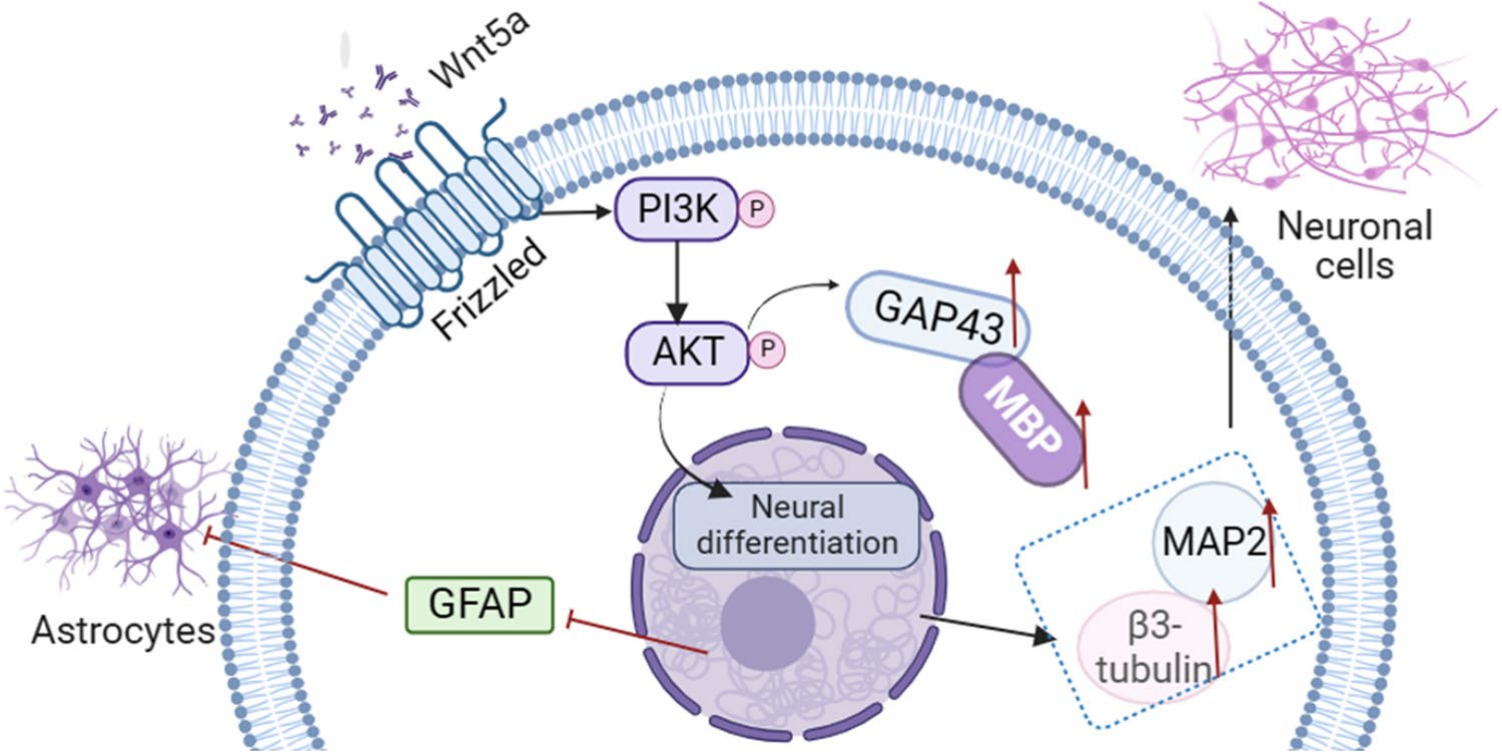
Illustration of the mechanism by which Wnt5a promotes neuron-directed differentiation of BMSCs through the PI3K/AKT signaling pathway

This study demonstrated that the presence of Wnt5a in BMSC may have a favorable impact on neuronal development (Fig. 6) by promoting neurite proliferation while reducing the population of astrocytes. This effect may be mediated through the PI3K/AKT signaling pathway but further studies are required to confirm this. Additionally, the transplantation of Wnt5a-modified BMSCs may have the potential to improve tissue repair and facilitate motor function recovery following SCI, emphasizing the viability of Wnt5a-modified BMSCs as a promising therapeutic intervention for SCI.

## Supplementary Information

ESM 1 (DOCX 11 kb)

ESM 2 (TIFF 1133 kb)

ESM 3 (XLS 4268 kb)

ESM 4 (XLS 165 kb)

## Data Availability

No datasets were generated or analyzed during the current study.

## Abbreviations

*SCI*: Spinal Cord Injury
*BMSC*: Bone marrow Mesenchymal Stem Cell
*SD*: Sprague Dawley
*FBS*: Fetal Bovine Serum
*PBS*: Phosphate-buffered Saline
*PI3K*: Phosphoinositide 3-kinase
*AKT*: Protein kinase B
*PCA*: Principal Component Analysis
*DEGs*: Differentially Expressed Genes
*RIPA*: Ristocetin-induced Platelet Aggregation
*BBB*: Basso-Beattie-Bresnahan
*H&E*: Hematoxylin and Eosin
*PFA*: Paraformaldehyde
*SEM*: Standard Error of Measurement
