# Supplementary material for: Transplantation of Wnt5a-modified Bone Marrow Mesenchymal Stem Cells Promotes Recovery After Spinal Cord Injury via the PI3K/AKT Pathway
Source: Mol Neurobiol. 2024 May 25;61(12):10830–44. doi: 10.1007/s12035-024-04248-8 (PMC11584464; doi:10.1007/s12035-024-04248-8)
Supplement: Supplementary file 1 — (DOCX 11 kb) [file 12035_2024_4248_MOESM1_ESM.docx]

**Primer sequence of Wnt5a**

Forward Primer

Name: 56 deg 1-27 Len:27 Score:74*

Predicted Melting Temperature: 56 degrees Celsius

GeneTool Score: 74

Start: 1 End: 27 Length: 27

Bases: 5'ATGAAGAAGCCCATTGGAATATTAAGC 3'

Reverse Primer

Name: 60 deg 1114-1143 Len:30 Score:75*

Predicted Melting Temperature: 60 degrees Celsius

GeneTool Score: 75

Start: 1114 End: 1143 Length: 30

Bases: 5'CTATTTGCACACGAACTGATCCACAATCTC 3'

Wnt5a-BamHIF: 5'cgcggatccgccaccATGAAGAAGCCCATTGGAATATTAAGC 3'

Wnt5a-NotIR: 5'ataagaatgcggccgcCTATTTGCACACGAACTGATCCACAATCTC 3'

**Gene sequence of Wnt5a**

atgaagaagcccattggaatattaagcccaggagtggctttggggaccgctggaggtgccatgtcttccaagttcttcctaatggctttggccacatttttctccttcgcccaggttgtaatagaagctaattcttggtggtccctaggtatgaataaccctgttcagatgtcagaagtatacatcataggagcacagcctctctgcagccaactggcgggactttctcaaggacagaagaaactctgccacttgtatcaggaccacatgcagtacattggagaaggcgcgaagacgggcatcaaagagtgccagtaccagttccggcatcggagatggaactgcagcacagtggacaacacttctgtctttggcagggtgatgcaaataggcagccgagagacagccttcacgtacgcggtgagcgctgctggagtggtaaatgccatgagccgagcatgtcgggaaggcgagctgtctacctgtggttgcagccgcgcagcacgccccaaggacttacctcgggactggctgtggggcggttgcggggacaacatcgactatggctaccgcttcgccaaggaattcgtggacgcacgagaaagggaacgaatccacgccaagggctcctatgagagcgcacgcatcctcatgaacttgcacaacaatgaagcaggtcgcaggacagtatacaacctggcagatgtagcctgtaagtgccatggagtgtctggctcctgtagcctcaagacatgctggctgcagctggcggacttccgcaaggtgggcgatgccctcaaggagaagtatgacagcgcagcggccatgaggttgaacagccggggcaagctggtacaggtcaacagccgcttcaactccccaaccacgcaggacctggtctacatcgacccaagtccggactactgtgtgcgcaacgagagcactggctcactgggcacgcagggacgcctgtgcaacaagacctcagaggggatggacggctgcgagctcatgtgctgtgggcgtggctatgaccagtttaagacggtgcagaccgaacgctgccattgcaagtttcactggtgctgctatgtcaagtgtaaaaagtgcacggagattgtggatcagttcgtgtgcaaatag
