# Supplementary figures and images for: Transplantation of Wnt5a-modified Bone Marrow Mesenchymal Stem Cells Promotes Recovery After Spinal Cord Injury via the PI3K/AKT Pathway
Source: Mol Neurobiol. 2024 May 25;61(12):10830–44. doi: 10.1007/s12035-024-04248-8 (PMC11584464; doi:10.1007/s12035-024-04248-8)

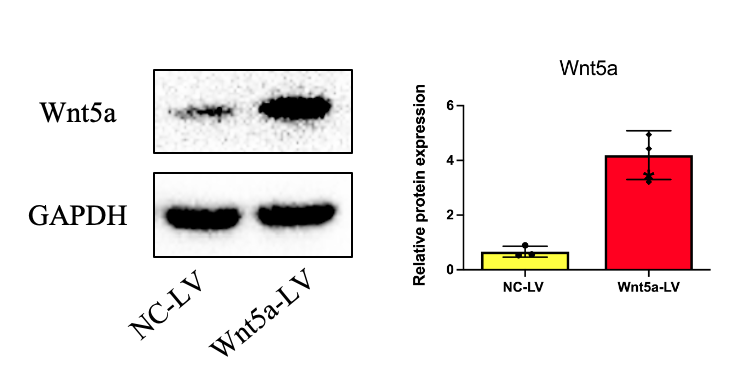

Supplement: Supplementary file 2 — (TIFF 1133 kb) [file 12035_2024_4248_MOESM2_ESM.tiff]
